# Physiology and technology for the ICU in vivo

**DOI:** 10.1186/s13054-019-2416-7

**Published:** 2019-06-14

**Authors:** Can Ince

**Affiliations:** Department of Intensive Care, Erasmus MC, University Medical Center, Rotterdam, ‘s-Gravendijkwal 230, 3015 CE Rotterdam, the Netherlands

## Abstract

This paper discusses the physiological and technological concepts that might form the future of critical care medicine. Initially, we discuss the need for a personalized approach and introduce the concept of personalized physiological medicine (PPM), including (1) assessment of frailty and physiological reserve, (2) continuous assessment of organ function, (3) assessment of the microcirculation and parenchymal cells, and (4) integration of organ and cell function for continuous therapeutic feedback control. To understand the cellular basis of organ failure, we discuss the processes that lead to cell death, including necrosis, necroptosis, autophagy, mitophagy, and cellular senescence. In vivo technology is used to monitor these processes. To this end, we discuss new materials for developing in vivo biosensors and drug delivery systems. Such in vivo biosensors will define the diagnostic platform of the future ICU in vivo interacting with theragnostic drugs. In addition to pharmacological therapeutic options, placement and control of artificial organs to support or replace failing organs will be central in the ICU in vivo of the future. Remote monitoring and control of these biosensors and artificial organs will be made using adaptive physiological mathematical modeling of the critically ill patient. The current state of these developments is discussed.

## Introduction

Personalized medicine for critically ill patients has gained much interest in recent years, mainly as a response to the lack of efficacy of randomized controlled trials as a vehicle for improving the treatments and outcomes in critically ill patients. Based on developments in cancer research, personalized medicine has been centered around genomics, biomarkers, and information obtained from large data sets [1, 2]. We questioned whether this approach was suited for critically ill patients, because of the rapidly changing condition of these patients and the complexity and heterogeneity of the pathophysiology of critical illness. Thus, we suggested that personalized medicine applied to critically ill patients should focus on the physiological condition of the patient integratively encompassing organ and cellular systems, a concept we called “personalized physiological medicine” (PPM) [3]. To this end, monitoring the direct environment of the failing organ and its cellular constituents continuously over time is necessary. Currently, such monitoring involves intermittent sampling, usually of surrogates of organ and cellular function; what is needed for the future will be focused on the microcirculation and the parenchymal level of the different organ systems. The requirement of continuous monitoring at the local level will require ex vivo diagnostic tools to be located in vivo. This requirement also holds for therapeutic modalities to support organ function now located ex vivo (e.g., mechanical ventilation, ECMO, and CVVH) to be developed for in vivo use (e.g., cardiac assist devices, artificial kidneys, and lungs). These concepts have led to the identification of the requirements needed for an ICU in vivo [4].

## Personalized physiological medicine

**Fig. 1:**
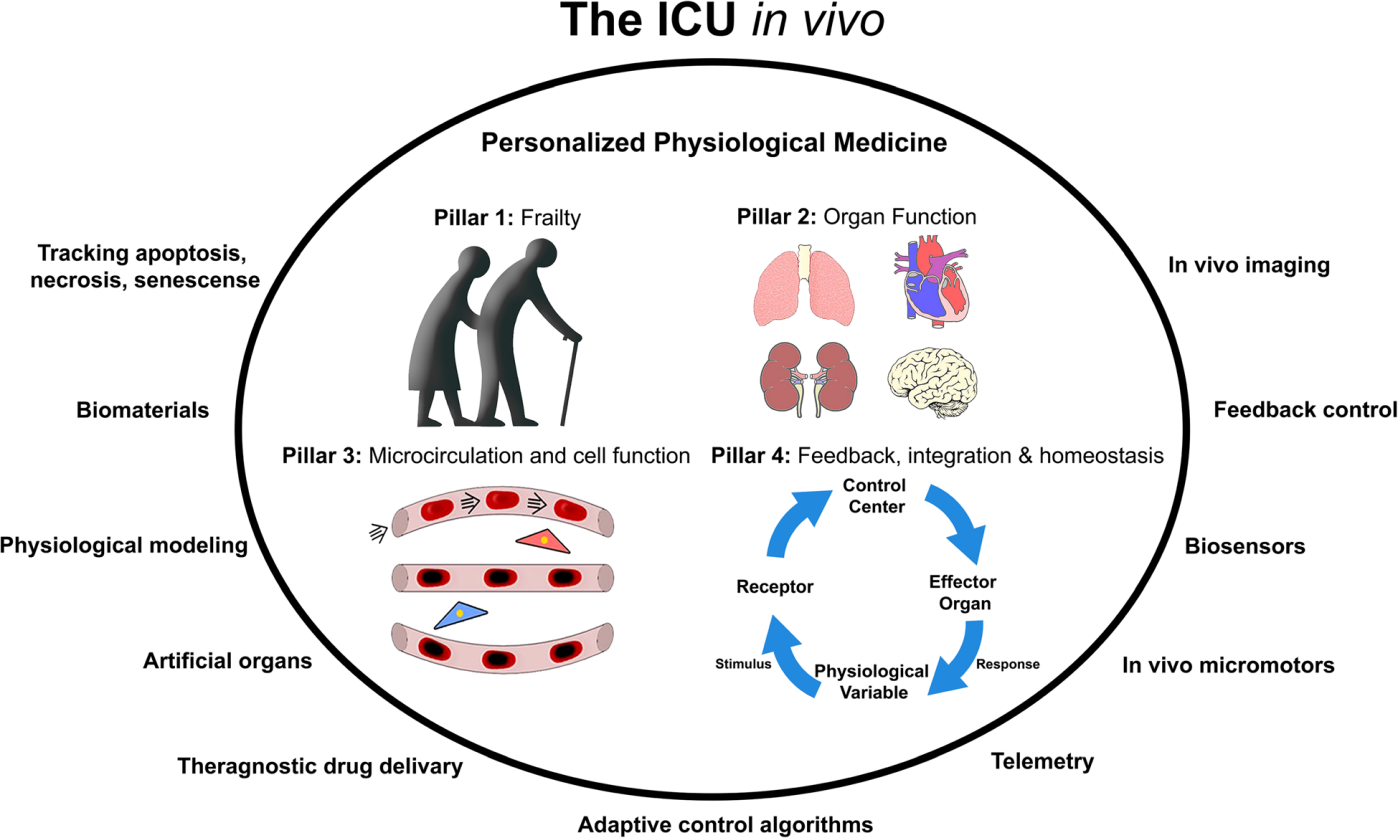
The ICU in vivo: This conceptual figure shows the physiological and technological requirements of the anticipated future of the ICU, where diagnostic devices and organ-assist devices now situated ex vivo will be transferred in vivo. Such an ICU in vivo will be based on personalized physiological tracking and control of bodily, organ, and cellular functions in a continuous manner. These are based on the four pillars of personalized physiological medicine (shown above) where assessment and (1) control of frailty and fitness, (2) organ function, (3) microcirculatory and cellular function, and (4) integration of information of the patient as a whole into a learning environment to provide feedback control of therapeutic modalities of the critically ill patient [3]. The technological components will have to be achieved to realize this concept of the ICU in vivo

A full understanding of the physiological state of the critically ill patient requires an integrative functional evaluation of the patient as a whole, from their organs to their microcirculation and ultimately to the functional activity of their parenchymal cells. A key feature for such a diagnostic and therapeutic platform is the requirement of providing continuous feedback regarding the functional state of the various compartments being monitored [5]. For such a PPM to be realized four main pillars can be defined (Fig. 1). Lack of fitness, the evaluation of frailty, and physiological reserve define the first pillar since these are directly related to cellular frailty underlying organ dysfunction. The pandemic nature of lack of fitness currently present in modern life-style is a major contributing factor to morbidity and mortality [6], and its identification and evaluation is important in understanding efficacy and prognostic impact of therapy [7]. Muscle dysfunction and atrophy are directly related to frailty and have been identified as important factors underlying organ failure [8]. Recent insights into the importance of exercise has identified muscle as being a key hormone-generating organ, releasing a family of hormones called myokines that are beneficial to a wide range of physiological functions [9]. These insights have opened a therapeutic window in terms of exercising muscle in critically ill patients, possibly improving outcomes [10]. The second pillar of PPM concerns the need to continuously assess the function of each organ individually as well as interactions among organs and with physiological compartments within the various organ systems to take into account the range and complexity of disease states underlying critical illness. To achieve this, technology will have to be developed and applied for monitoring of physiological biomarkers in advance of changes in pharmacological biomarkers as early indicators of impending organ dysfunction [11]. From this perspective, every therapeutic intervention, whether it be the administration of fluids, inotropes, or blood, is an opportunity to evaluate the functional state and physiological reserve of the various organ systems. Pillar three concerns assessment of the microenvironment of the organs, consisting of the microcirculation (oxygen and perfusion) and ultimately parenchymal function of the organs in response to disease and therapy. A step toward the realization of pillar 3 is the current interest in hand-held vital microscopes (HVM) as bedside tools to assess the microcirculation, providing complimentary information to conventional systemic variables concerning the nature of critical illness [12]. HVM can also be used effectively to evaluate microvascular reactivity and physiological reserve [13]. Use of integrative evaluation of various physiological variables to identify the state of disease and response to therapy from organ to cell in vivo as well as control of implantable organ will require placement of biosensors in vivo with wireless ex vivo communication. Such information will need to be integrated in mathematical models of the patient for closed-loop control of therapeutic interventions. Therefore, integration and feedback characterize pillar 4 in much the same way as physiological homeostasis does.

## The cell in distress

The ultimate success of the cardiovascular system to ensure parenchymal health in supporting organ function is to ensure adequate delivery of oxygen and nutrients via the microcirculation to parenchymal cells. Normal physiological injury resulting in cell dysfunction is resolved by cell clearance and tissue repair. If injury is too severe for the support of organ dysfunction by endogenous rescue and repair mechanisms, critical care support is indicated. In the ideal case, continuous information regarding the functional state of the parenchymal cells responsible for organ function should be available for optimal therapeutic control. Since this is currently not possible, such information is indirectly obtained by intermittent assessment of surrogates of organ function including pharmacological biomarkers of organ dysfunction. For rapidly changing systems such as those in critical illness, constraints are imposed by the Nyquist-Shannon sampling theorem, whereby the variable responsible for control of a system has to be sampled at least twice the highest rate of change of the system; this requires, almost by definition, continuous monitoring [14, 15]. Thus, if cellular dysfunction underlies organ failure, monitoring biosensors would have to be developed in close proximity to the parenchymal cells of the organ systems to obtain precise knowledge of the determinants of cellular dysfunction in a continuous manner. Nevertheless, knowledge concerning these determinants needs to be acquired first. A discussion of the several cellular processes being compromised as a result of injury, however, is beyond the scope of this paper; nevertheless, a brief discussion of the cellular process leading to cell death is relevant in context of the ICU in vivo. Such information is needed to design accurate ICU in vivo biosensors that are able to identify cellular frailty and cells at risk for dying.

The best-known form of cell death is necrosis caused by injury from external factors such as trauma, ischemia, and infection. Necrosis results in a rapid permeabilization of cell membranes so that cellular constituents spill into the extracellular space. This causes inflammatory activation that may fuel systemic inflammation or may contribute to an adaptive response to resolve the deleterious effects of necrosis [16]. Necroptosis is a programmed form of necrosis that also leads to rapid cellular permeabilization, spilling of cellular constituents, and damage-associated molecular patterns eliciting an immune response [17, 18]. Such an immune response can elicit a storm of cytokines that can, as in necrosis, itself cause tissue injury [19]. In contrast to necrosis, apoptosis is a catabolic process where cellular constituents are packaged into membranes called apoptotic bodies and are phagocytosed by macrophages. These apoptotic bodies are immunologically silent and can be thought of as a physiological method of replenishing dysfunctional cells and disposing of aged cells. Its activation is considered to be an important physiological response associated with recovery from critical illness [20, 21]. Programmed cell death of frail or injured cells can also contribute to organ dysfunction. For example, programmed cell death of red blood cells referred to as eryptosis, precedes hemolysis, leading to release of free Hb, causing parenchymal damage that contributes to anemia and microcirculatory alterations [22]. Furthermore, endotoxin can lead to endothelial apoptosis resulting in vascular barrier compromise that fuels organ dysfunction [23].

Cell senescence is a different type of cell dysfunction and is associated with frailty in old age and thought to underlie a wide range of disease states, including critical illness [24]. Cellular senescence has historically been viewed as an irreversible cell-cycle arrest mechanism that acts to protect against cancer [25]. However, in aging, cell senescence is upregulated, thus contributing to a decline in the ability of tissue repair and regeneration [26]. Such senescent cells have been referred to as zombie cells and are currently under investigation as a target for achieving longevity [27]. Its identification fits well into the first pillar of PPM, namely, the identification of frailty. Recent interest in cell cycle arrest biomarkers as markers of the presence of acute kidney injury (AKI) indicates the link between cell senescence and organ failure [28]. Such biomarkers identify the presence of senescent tubular cells that are thought to contribute to the proliferation of non-functional fibroblasts resulting in fibrotic kidneys defining the progression of AKI to chronic kidney disease [29].

Endogenous mechanisms such as phagocytosis are present for clearing apoptotic, necroptotic, and necrotic cells, and these processes are central to ensuring health and maintaining organ function following injury. Autophagy is a programed mechanism for cell clearance by the formation of autophagosomes and autolysosomes, where intracellular compounds are degraded. In sepsis, there is an initial upregulation of autophagy as defense of the septic insult [30] but followed by downregulation of cell apoptosis and its subforms such as mitophagy may contribute to organ dysfunction [31–33]. The current thinking is that knowledge of necrosis, apoptosis, and autophagy may be interactive in determining the pathway that is most beneficial for survival [34].

Therefore, it is expected that monitoring of necrosis, necroptosis, senescence, and apoptosis will identify whether a therapeutic strategy will lead to organ regeneration or if organ support or replacement by artificial devices is warranted. Due to the spatial and temporal heterogeneity of such cellular dysfunction, interactive biosensors will be required to be close to parenchymal cells in a continuous manner to provide information necessary to support clinical decisions and controlling drug delivery and organ-assist devices. The understanding of technology in the context of the above is viewed not only as devices but to the whole continuum of external from artificial hardware to biologics and any methodology used as a therapeutic intervention including drugs.

## Theragnostics and biosensors for the ICU in vivo

The current diagnostic modalities lack the ability to provide continuous and quantitative information about specific organ locations. With regard to drug therapy, there is a need for more precise homing of therapeutic drugs and a need to know whether the drug has achieved its delivery and accomplished its therapeutic action, an approach referred to as theragnostic drug delivery. Its technological implementation, in which feedback is given regarding arrival and therapeutic action, will require the development of new generations of nanoparticles composed of homing devices, communication modalities, and controlled mobility. Such nanoparticles are currently mostly being developed in the field of oncology [35, 36]. New generations of materials are being explored, including polymeric, gold, and silica for development of theragnostic and biosensor platforms, including the highly promising carbon nanotubes (CNT). CNTs have unique electrical, physical, and chemical properties particularly suited for the engineering requirements of personalized nanomedicine [37, 38]. In addition to these properties, they also possess specific semiconductor properties that allow implementation of electronics superior to those of conventional silicon-based electronics [39, 40]. Biomolecules derived from cells, bacteria, and viruses can also be incorporated into the electronic environment of biosensors, an approach referred to as synthetic biology [41]. In this way, engineered bacteria can be used to home in on tumors and emit signals when successfully reaching their target and achieving their therapeutic actions [42].

Biosensors and drug delivery systems for the ICU in vivo will require integration of biosensors, wireless communication, and power sources. Such wireless biosensors are currently under development for monitoring body surfaces’ measurement of body fluid compositions [43]. Wireless control of nanomotors incorporating biosensors is another field that is relevant for the ICU in vivo [44]. Currently, mobile imaging biosensors have been in clinical use, including wireless video capsules as an alternative to gastric endoscopy, where control of intragastric movement and location can be achieved by external application of magnetic fields [45]. It is not inconceivable that nano-imaging modalities will be developed for future intravascular imaging of the microcirculation and their cells (Pillar 3 of PPM) in much the same way as handheld vital microscopes are currently being used to obtain cellular information about the microcirculation [12].

## Artificial organs in the ICU in vivo

Tissue engineering is a rapidly developing area, where engineering, biology, and medicine are interacting to make functional organs [46]. For identification of failed cellular systems unable to support organ function and cannot be therapeutically rescued, organs will either have to be supported or replaced by artificial devices. Currently, organ assist devices such as mechanical ventilation, ECMO, cardiac assist devices, and CVVH are applied ex vivo. However, in the ICU, in vivo artificial organs will be placed in vivo for permanent support or replacement. Current focus of the engineering of artificial organs and/or assist devices for failing hearts and kidneys for in vivo integration are under development (e.g., [47, 48]). Tissue engineering platforms under current investigation include the use of organ extracellular matrixes [49, 50] and even 3D-printed organs [51]. These tissue-engineered organs and assist devices will require integration into normal physiology to achieve their functional role in maintaining homeostasis. This will form a major challenge for the ICU in vivo intensivist.

## Feedback and integration in the ICU in vivo

The fourth pillar of PPM involves integration and feedback and is based on engineering concepts related to control theory [52]. Linking mechanical ventilation to ECMO in a closed-loop manner is a current example of clinical implementation of feedback [53]. It is anticipated that closed-loop systems for therapeutic and organ control will be central in the ICU in vivo. Wireless communication between sensor and effectors will be an important pre-requisite for implementation of such closed-loop systems in much the same way as is physiologically the case for homeostasis (Fig. 1). Signals from biosensors in vivo could control theragnostic drug delivery and artificial organs. Such signals will generate a massive amount of information and will need to be evaluated to extract essential parameters needed for control. Such evaluation will require the use of mathematical models of the various functional activities of organs and parenchymal systems in an integrative and continuous manner. Currently, such integrative modeling is being developed using a methodology referred to as computational physiology under the banner of the International Union of Physiological Sciences [54]. This Physiome Project will have to be extrapolated to model the critically ill patients, taking into consideration their ever-changing condition, and will require self-learning models, including artificial intelligence such as being developed in various areas of critical care medicine [55, 56]. It is expected that such a virtual mathematical model of the individual patient will be the platform whereby the intensivist will be able to steer the patient from critical illness into recovery.

In conclusion, there are many challenges to be overwon concerning the practical implementation of the concepts discussed in this paper for the establishment of the ICU in vivo. Most importantly, despite critical care medicine being a multi disciplinarian science, much more participation of disciplines outside intensive care medicine is needed. This is especially a challenge for physiologists who unfortunately, and I say this as a physiologist, have shown a marginal interest in the pathophysiology of intensive care medicine and have chosen for a reductionist approach to pathophysiology [57]. But also engineers who have limited themselves to technical aspects of intensive care medicine should extend their interests into the clinical issues of complexity of living systems and how these come under threat during critical illness. And finally, the intensivists themselves must be even more willing to embrace new technologies and concepts in moving intensive care medicine forward instead of demanding yet another RCT. Success will be guaranteed if the clinical, scientific, and engineering community will come together to meet this challenge for the future ICU in vivo.
